# Geographic Altitude, Ocular Diseases and Injuries

**Published:** 2014

**Authors:** Rovshan M Ismailov

**Affiliations:** Complex Mechanisms of Disease, Aging and Trauma (CMDAT) Research Foundation, Glendale, CO 80246, United States.

**Keywords:** Ocular Diseases, Injuries, Geographic Altitud

Various ocular disorders remain a major concern for medical care providers and health policy makers. Only in the US more than 6.6 million people were affected by blindness in 2011 (1). Meanwhile, even though people mostly inhabit low or moderate altitudes, about 140 million people worldwide live above 2500 meters (2). Therefore, association between various vision disorders and high altitude remains a significant health concern for millions of people throughout the world.

Residents of high-altitude areas are affected by reduced oxygen supply conditions. This, on one hand, results in higher levels of a hypoxia-inducible factor 1 (HIF-1), which increases expression of vascular endothelial grow factor (VEGF). In turn, this promotes pathological neovascularization and subsequent deterioration of such eye conditions as age-related macular degeneration (AMD) (3). On the other hand, high-altitude hypoxia triggers the release of erythropoietin (EPO), a general tissue-protective cytokine which was shown not only to protect neurons following retinal ischemia in vivo, but also to improve their recovery following injury (4). Other findings supported the hypothesis that EPO can be used to treat atrophic AMD (5). EPO administered systemically was shown to protect retinal ganglion cells somata from an optic nerve crash (6).

EPO was also found to be effective in treating various sensorimotor disorders in a rat model of diffuse traumatic brain injury (TBI) and, very importantly, these beneficial effects occurred only under hypoxic conditions (7). Finally, EPO has a wide range of favorable effects in diabetic retinopathy, such as direct protection of retinal neuronal cells and the blood-retina barrier function (8). Those coupled with complex anti-inflammatory and anti-oxidant effects exerted by EPO as well other beneficial properties described above could potentially impact the overall epidemiology of various ocular disorders as well as non-refractive vision impairment.

Undoubtedly, further studies are needed to estimate the prevalence of various ocular disorders at different altitude levels. Currently, however, no population studies have examined this issue. Moreover, I hypothesize that not only the prevalence, but also prevention, treatment and rehabilitation of various forms of vision and ocular disorders can be influenced, in part, by geographic altitude and the major mechanism is the excessive release of EPO in the high-altitude area.

A special consideration should be placed on traumatic and war-related injuries (i.e. blast-related ocular trauma) that occur in the high-altitude level. Ocular injuries as well as trauma to other anatomical structures related to vision (i.e. optic nerve) are often viewed as accidental mechanical impact with prevalence that cannot be influenced by internal substances such as hormones (i.e. EPO). However, ocular and adjunct structures’ injury is complex entity with several pathophysiological events such as ischemia from vasospasm, apoptosis, inflammation, etc. An increased EPO production in the high-altitude is likely to affect many of these stages (i.e. cellular apoptosis). Therefore, not only the prevalence of ocular and optic injuries, but also various complications following trauma (i.e. vision impairment) are likely to be influenced by geographic altitude.

Why is this important? Evidence that prevalence of important vision and ocular disorders, ocular injuries and their outcomes may vary at various altitude areas may open up new avenues for treatment, including emergency ocular trauma and optic nerve injury care. Such studies may help to identify various factors (i.e. age, gender, etc.) related to prevention, treatment and rehabilitation of patients diagnosed with a number of vision and ocular disorders at various altitudes. In addition, evidence that the prevalence of some vision and ocular disorders as well as ocular injury outcomes is lower in the high-altitude area would help to identify a new line of treatment with similar effect such as various EPO regimens, etc. Another research direction is to clarify the role of hyperbaric treatment as well as its possible complications in the prevention, treatment and rehabilitation of various vascular pathologies. It may also help to establish new research directions in this area as well as contribute to a better understanding of the pathophysiological mechanism of certain complex eye conditions (i.e. optic nerve injury, vision complications following TBI), which are currently not well understood. Since, on one hand, wartime theaters of operations have occurred in various parts of the Earth and very often much above the sea level and, on the other hand blast-related ocular trauma is one of the most common injuries encountered by military combat today it may also help to improve the initial treatment of ocular trauma, optic nerve injury as well as vision and ocular disorders following TBI from the point of impact (i.e. blast) to the point of medical evacuation (9). Furthermore, there is a need to examine the relationship between various ocular pathology and both acute and chronic exposure to high-altitude. This is of particular importance since a short, more likely than a long-term hypoxia, has been hypothesized to promote the protection to neuronal cells (10). Finally, knowledge of geographic altitude increasing the risk of certain ocular conditions may help physicians and health policy makers to identify new at risk groups.
